# Clinical Trials Conducted on Herbal Remedies for the Treatment of Melasma: A Scoping Review

**DOI:** 10.1111/jocd.16741

**Published:** 2024-12-22

**Authors:** Mohammad Mahdi Parvizi, Maryam Hekmat, Nahid Yousefi, Rojan Javaheri, Arman Mehrzadeh, Nasrin Saki

**Affiliations:** ^1^ Molecular Dermatology Research Center Shiraz University of Medical Sciences Shiraz Iran; ^2^ Research Center for Traditional Medicine and History of Medicine Shiraz University of Medical Sciences Shiraz Iran; ^3^ Vice Chancellor of Academic Affairs Smart University of Medical Sciences Tehran Iran; ^4^ Department of Dermatology, Molecular Dermatology Research Center Shiraz University of Medical Sciences Shiraz Iran; ^5^ Student Research Committee, School of Medicine Shiraz University of Medical Sciences Shiraz Iran

**Keywords:** clinical trial, complementary therapies, herbal medicine, melanosis, Persian medicine, review

## Abstract

**Introduction:**

Melasma, also known as chloasma, is a common skin disorder characterized by acquired hyperpigmentation. Many patients with this condition prefer using herbal remedies instead of chemical agents. This study aims to review clinical trials conducted on the effectiveness of herbal remedies in treating melasma.

**Methods:**

In this scoping review, we searched the PubMed, Scopus, Web of Science, Cochrane, SID, and Magiran databases until August 2024. We designed the search strategy using MeSH database keywords “melanosis,” “herbal medicine,” “plant extracts,” “complementary therapies,” “traditional medicine,” “Persian medicine,” “clinical trials,” and their Entry Terms. We then reviewed and summarized the relevant articles.

**Results:**

We found a total of 21 clinical trials examining the effectiveness of herbal remedies in treating melasma. The literature review revealed that licorice, rhubarb, a mixture of melon seed and chickpea, sorrel, *Aloe vera* leaf gel, parsley, tomato, fern, olive, pine bark, and Indian gooseberry had positive effects in treating melasma. Licorice is the most extensively studied herbal remedy for melasma treatment. Some patients who used licorice, rhubarb, and parsley experienced redness and skin allergies.

**Conclusion:**

Few studies have evaluated the effectiveness of herbal remedies in treating melasma. Further research, including clinical trials, systematic reviews, and meta‐analyses, is necessary to assess the efficacy of herbal remedies and natural products, as well as their potential adverse effects.

## 1 Introduction

Melasma, or chloasma, is a common skin disorder characterized by acquired hyperpigmentation [1, 2]. It presents as irregular, symmetrical patches on the skin, ranging in color from reddish‐brown to blue‐gray, and can vary in size from small spots to larger, darker patches [2, 3]. Melasma primarily affects areas of the body that are more exposed to sunlight, with the face being the most commonly affected area [4]. Overall, about 1% of people in the general population have melasma. However, that number can rise to 9% to 50% in high‐risk groups [2]. Geographical and racial variations among individuals in different countries can account for these differences [1]. Women and individuals with darker skin tones are more likely to experience melasma [4].

The exact cause of melasma remains unknown; however, genetics and environmental factors are believed to play significant roles in its development [5, 6]. Genetic predisposition and family history are considered the most important factors contributing to melasma. Approximately 50% of melasma patients report a family history of the condition [7]. Additionally, melasma is more prevalent in women compared to men, with a relatively low incidence in males [8, 9].

Hormonal changes and exposure to ultraviolet (UV) radiation are key factors influencing melasma [4, 8]. Studies have strongly correlated the development of melasma with increased sunlight and UV radiation exposure [5, 10]. UV radiation and sunlight induce the production of free radicals through lipid peroxidation in cell membranes. These free radicals stimulate melanocytes to produce more melanin, leading to the formation of melasma and the darkening of the skin [11].

Several studies have proven the effects of hormones on the incidence of melasma [12, 13]. Pregnant women have a significantly higher risk of developing melasma compared to others. While the exact mechanism of melasma occurrence during pregnancy remains unknown, we have observed a natural increase in levels of estrogen, progesterone, and melanocyte‐stimulating hormones [13]. Additionally, endocrine diseases like thyroid disorders can also impact the incidence of melasma. Researchers have found a fourfold increase in melasma occurrence in patients with thyroid disorders [14]. Some medications, including cosmetic agents that increase the sensitivity of the skin to the sun and anticonvulsants, can also contribute to the development of melasma [15].

Melasma is typically categorized into four types based on the pigment pattern: epidermal (on the skin), dermal (subcutaneous), mixed, and type IV (most commonly seen in individuals with very dark skin color) [16].

Diagnosing melasma is usually straightforward in clinics due to visible symptoms such as brown spots on the skin [5, 17]. A more detailed examination using Wood's lamp light (300 to 400 nm) can confirm the diagnosis. Laboratory studies are generally not necessary for diagnosing this disease [17].

Despite the existence of effective treatments for controlling and improving melasma‐induced spots, treating this disease still presents a multitude of challenges. The use of topical hydroquinone cream, along with avoiding exposure to sunlight and estrogenic hormones, appears to be the best treatment option for these patients [17, 18]. Sunscreen products play a crucial role in the treatment of melasma, with sunscreens having an SPF greater than 50 showing significant effectiveness [17]. Sunscreens containing hydroquinone are particularly effective in preventing and treating melasma in susceptible individuals [19].

Nowadays, the first line of treatment for melasma involves the use of a topical combination cream that includes 4% hydroquinone, 0.05% tretinoin, and fluocinolone acetonide ointment 0.01% [19]. Additionally, studies have found that creams, lotions, and gels containing corticosteroids and tretinoin can help clarify skin discoloration [18, 19]. Other treatment options to reduce skin melanin include chemical skin peeling using glycolic acid and salicylic acid, laser therapy, and dermis super inflation [19]. However, it is important to note that some of these treatment methods, especially laser therapy, can lead to complications such as epidermal necrosis, increased pigmentation after inflammation, and hypertrophic scarring [20, 21]. Therefore, they should only be considered if the first line of treatment fails. In cases where complications are absent, second‐line treatment methods generally result in faster recovery compared to first‐line drugs [17, 19].

Due to the side effects associated with chemical drugs, such as skin inflammation and redness, as well as the bothersome skin stains caused by melasma, many patients have turned to herbal remedies for treatment [22, 23]. Surprisingly, there has been a lack of comprehensive reviews of clinical trials conducted on herbal remedies for melasma treatment. We aim to focus this scoping review on the efficacy of herbal remedies examined in clinical trials for melasma treatment.

## 2 Methods and Material

### 2.1 Study Design

This scoping review study aims to investigate the efficacy of herbal remedies in treating patients with melasma. For preparing this document, we used the PRISMA Extension for Scoping Reviews (PRISMA‐ScR) guideline.

### 2.2 Eligibility Criteria

We searched for relevant clinical trials using keywords, including “melanosis,” “herbal medicine,” “plant extracts,” “complementary therapies,” “traditional medicine,” “Persian medicine,” “clinical trials,” and their synonyms from Entry Terms of MeSH databases. The search was conducted without any time limit up until August 2024. We also excluded case reports, reviews, and letters to the editor.

### 2.3 Information Sources

The databases include PubMed, Scopus, Web of Science, Embase, Cochrane, and Persian‐language databases like SID and Magiran. Moreover, we used the Google Scholar search engine to complete our search process.

### 2.4 Search and Selection of Sources of Evidence

After creating the search syntax, two authors, M.M.P. and N.Y., participated in screening and selecting relevant articles from the extracted articles from the databases. This was done by reviewing the titles and abstracts of the articles.

### 2.5 Data Charting Process and Synthesis of Results

In the data charting process, M.M.P., N.S., R.J., and M.H. were involved in reviewing the selected articles, data extraction, and confirmation of the data. Then, M.M.P., R.J., A.M., and M.H. tried to summarize and organize the extracted data using a researcher‐made tabulated form.

### 2.6 Data Items

Documentation included details about the type of clinical trial, the plant used, the drug application method, the drug form, participant details, the outcomes and end‐point assessment tools, the study results, and any reported adverse effects of the prescribed medications.

### 2.7 Ethics Statement

The protocol for this study received approval from the Research Ethics Committee of Shiraz University of Medical Sciences (Ethics Code: IR.SUMS.MED.REC.1399.597).

## 3 Results

The search strategy led to the selection and review of 19 clinical trials, as shown in Table 1. The following section discusses the studied plants and the results of each article separately.

**TABLE 1:** The clinical trials conducted on herbal remedies for the treatment of melasma.

Authors (year)	Type	Total samplesize	Age (year)	Gender	Type of melasma	Herbal remedy	Form of prepared	Dosage	Control group	Follow‐up duration	Tools assessment	Outcome	Side effect
Hossein Matourian pour (2010) [24]	Clinical trial	27	17–47	27F	Malar melasma	Licorice	Cream 4%	Licorice cream 4% Once a day	4% hydroquinone cream	8 W	mMASI score	Based on mMASI score the licorice extract has less effect than 4% hydroquinone	No complications occurred in the licorice 4% group
Mohamed Amer et al. (2000) [25]	Clinical trial	20	18–40	20F	Epidermal melasma	Licorice	Cream 2%	Licorice cream 2% twice a day	Placebo cream	4 W	Pigmentation intensity criteria	About 80% of the lesions treated with licorice had excellent response. This rate was 0% in the control group.	Erythema and sensitivity were observed in two patients using licorice
Zhang Hui et al. (2019) [22]	Clinical trial	31	20–60	31	Melasma	Rhubarb	Liquid base	Rhubarb plant (liquid base) twice a day	Liquid placebo	8 W	Melanin and mexameter index	No significant difference was observed between placebo and rhubarb plant	In rhubarb group, two mild complications (skin allergy) were observed only
Marjan Mahjour et al. (2020) [26]	Clinical trial	32	> 18	32F	Melasma	Melon seeds and chickpeas *Cicer arietinum* L. and *Cucumis melo* seeds	Cream	Combination of melon seeds 5% and chickpeas 5% on a daily basis and 0.5 units of finger's head	4% of hydroquinone	12 W	MASI score	Both groups recovered significantly	No side effects
Clarisse G. Mendoza et al. (2014) [27]	Clinical trial	45	18–60	45F	Epidermal and mixed melasma	Sorrel or Rumex Occidentalis	Cream	Sorrel cream 3% twice a day	4% of hydroquinone	8 W	MASI score	The reduction of pigmentation in mexameter and the decrease of MASI score were higher in patients in the hydroquinone group, but in the 8th week, the improvement rate of the sorrel group was significantly higher than the other group	No side effects
Farwa Naqvi et al. (2020) [28]	Clinical trial	120	20–50	91F, 17M	Melasma	*Aloe vera* leaves	Gel	*Aloe vera* leaf gel every night	Phenylalanine gel 2%	12 W	MASI score	The reduction of the MASI score was not significant in patients treated with *Aloe vera* gel, while the group treated with phenylalanine significantly improved	No side effects
Simin Shamsi Maimandi et al. (2016) [29]	Clinical trial	40	20–40	40F	Melasma	Licorice	Cream	Licorice cream 4% twice a day	Placebo cream	12 W	mMASI score	The mMASI score in the intervention group decreased from 2.7 ± 11.03 to 0.6 ± 41.41 and in the control group decreased from 2.9 ± 11.25 to 1.2 ± 37.2	No side effects
Liza Afriliana et al. (2021) [30]	Clinical trial	62	30‐55	62F	Melasma	Tomato	Extract	Supplements extracted from tomatoes once a day	Placebo	12 W	mMASI score and rate of IL‐17	The results of this study showed that the group that used tomato supplements had significantly decreased pigmentation and mMASI score	No side effects
Shamshad Akram et al. (2013) [31]	Clinical trial	84	16–45	74F, 8M	Epidermal melasma	Licorice	Topical	Licorice 4% plus ascorbic acid 5% daily	Licorice 4%	8 W	MASI score	The group that had used ascorbic acid along with licorice had improved and the severity of melasma or MASI score had decreased significantly	No side effects
Chi‐Lyuk Guo et al. (2018) [32]	Clinical trial	40	25–65	37F, 3M	Melasma	Tropical fern or polypodium leucotomos	Capsule	Tropical fern supplement daily two or four capsules (240–480 mg)	Placebo (480 mg)	12 W	mMASI score and quality of life criteria	mMASI score and quality of life criteria improved significantly in patients receiving equatorial fern supplement	No side effects
Julia De Toledo Bagatin et al. (2020) [33]	Clinical trial	42	32–49	42F	Melasma	Olive	Oral and topical	Olive oil orally	Oral and topical placebo	90 Days	mMASI score	There was no significant difference between the three groups in terms of mMASI score	No side effects
Ahmed et al. (2013) [34]	Clinical trial	33	24–85	33F	Melasma	Tropical fern or polypodium leucotomos	Supplement	Tropical fern supplement (240 mg) three times a day	Placebo	12 W	melanin and MASI score	Despite the improvement of melanin and MASI score in both groups, there was no significant difference between the two groups	No side effects
Lima et al. (2020) [35]	Clinical trial	44	39 ± 7 years	44F	Melasma	Pine bark or maritime pine bark (pcnogenol)	Supplement	Pycnogenol supplement 75 mg twice daily	Placebo	60 Days	Criteria of mMASI score and quality of life in patients with melasma	According to the two criteria of mMASI score and quality of life, it seems that pyconogenol significantly improves these two factors	No side effects
Neda Bavarsad et al. (2020) [36]	Clinical trial	22	Average 33.5 ± 7.8 in placebo group and 34.7 ± 10.2 in the molten group	22F	Melasma	Tomato	Cream	Combination of 0.05% lycopene extracted from tomato and wheat bran 3.45% twice a day	Placebo	3 Months	MASI score	The patients in the control group and the intervention group were not different in the MASI score 1 month after discontinuation	No side effects
Adilson Costa et al. (2010) [37]	Clinical trial	56	18–60	56F	Epidermal melasma and mixed melasma	Indian gooseberry or emblica	Topical	Topical use of Indian gooseberry and licorice 7% twice a day	2% hydroquinone cream	60 Days	Pigmentation rate	No significant difference between the two groups in terms of disease improvement	No side effects
Hayra D Avianggi et al. (2022) [38]	Clinical trial	62	35‐55	62F	Melasma	Tomato	Oral	Oral tomato extract supplement (lycopene 30 mg) plus topical sunscreen and hydroquinone 4%cream	All subjects applied topical sunscreen and hydroquinone 4%cream	12 W	Superoxide dismutase (SOD), and MASI score	SOD level was higher in the treatment group and MASI score was also significantly decreased	No side effects
Penpun Wattanakrai et al. (2022) [39]	Clinical trial	25			Epidermal or mixed‐type	Milk thistle	Topical	Silymarin cream on one side	Hydroquinone on the contralateral side	3 months	Colorimeter by relative lightness index (RL*I), mMASI score, and patient self‐assessment	mMASI scores decreased after both treatments but only significant on hydroquinone side (17.97% reduction (*p* = 0.02) vs. 7.11% (*p* = 0.32) on the silymarin side) no significant difference of the RL*I and mMASI score between the 2 treatments, patient satisfaction was quite similar	
Shamsi et al. (2023) [40]	Clinical trial	55	20–55	49F, 6M	Epidermal	Lentil, bitter almond, and fig	Topical	Tila‐e‐Kalf (fine powder of lentil ( *Lens culinaris* ) and bitter almond (*Prunus amygdalus* var.amara) in equal proportion then mixed with the decoction of fig ( *Ficus carica* ) in to make a homogenous paste) once daily	Hydroquinone 4% cream	8 W	MASI score and, DLQI (Dermatology Life Quality Index), and PGA (Physician Global Assessment) and colored photographs	Similar improvement was noticed in all parameters	One patient had mild itching
Javedan et al. (2021) [41]	Clinical trial	49	Mean age of 32.18 ± 8.69	40F, 9M	Melasma	Dorema ammoniacum	Topical	6% *D. ammoniacum* gum combined with oserin in 10% ratio	Placebo	30 days	Mean melasma severity index (MMASI) score	Mean Melasma severity index (MMASI) score in the treatment group was significantly lower than before treatment (decreased from 86.98 ± 69.48 to 31.03 ± 32.62 (*p* < 0.05).)	No side effects
Bakhtyari et al. (2024) [42]	Clinical trial	110	> 40	110F	Female participants who were aged 15 years or older	Polyherbal Syrup Containing Lemon Balm, Damask Rose, and Fennel	Oral	Polyherbal Syrup Containing Lemon Balm, Damask Rose, and Fennel	Placebo	12w	MELASQOL, MASI score, Mexameter MX18 to measure Melanin and Erythema	Significant reduction in MASI score, melanin, pigmentation, MelasQOL	No obvious side effects
Kheirieh et al. (2024) [43]	Clinical trial	30	Mean age of 2.73 ± 6.98	30F	Female patients diagnosed with epidermal facial melasma	*T. chebula* 5% cream	Topical	*T. chebula* 5% cream	Hydroquinone 2% cream	12 W	mMASI score	mMASI score was significantly reduced in *T. chebula* group 4, 8, and 12 weeks after initiating the study.	No side effects

Abbreviations: MASI, Melasma Area Severity Index; mMASI, modified Melasma Area Severity Index.

### 3.1 Rhubarb

Zhang Hui and colleagues conducted a study on 31 melasma patients. The patients were divided into two groups: one group received a placebo, while the other group received rhubarb in liquid form. Patient satisfaction and improvement of melasma were evaluated in follow‐ups conducted at 4, 8, and 12 weeks. The patients took the drug twice a day for a duration of 8 weeks. The study found that patient satisfaction with rhubarb was 79.31%, whereas with the placebo, it was only 24.14%. The study also evaluated improvements in pigmentation and melanin index between the two groups. However, the results showed no significant difference between the placebo and rhubarb in terms of improving melasma spots. Interestingly, the melanin index increased again in the 8th week, despite a decrease in the 4th week. After completing the study, the melanin index was measured at 12 weeks, the results of which indicated a worsening of the melanin index (however, the melanin index decreased compared to the first day of the study). The deterioration of patients can be due to stopping sunscreen consumption after the end of the study. No significant complications were observed in either group [44].

Several studies have proven the anti‐inflammatory, anti‐scarring, and antioxidant effects of rhubarb. The plant's anti‐inflammatory and antioxidant properties seem to have a positive impact on sunlight‐induced melasma, especially at higher concentrations [44, 45]. However, further studies with larger sample sizes and the determination of the active ingredient of this plant are necessary to investigate its effect on melasma‐induced spots.

### 3.2 Chickpea

According to Persian medicine sources, chickpea is recognized as a skin lightening plant that can reduce skin lesions caused by melasma [46, 47]. The mechanism of action of this plant is inhibition of the tyrosinase enzyme and free radicals [29]. In a study, the effectiveness of a combination of chickpea and melon seed compositions was compared with the effect of 4% hydroquinone. The study evaluated the impact of these plants on melasma severity over a period of 12 weeks. The results demonstrated that this combination of drugs can significantly improve melasma‐induced spots without causing any serious side effects. Both groups showed significant improvement after 12 weeks [26].

### 3.3 Melon Seed

A recent study looked at how melon seed affects melasma and discovered that the anti‐tyrosinase properties of the seeds, when mixed with chickpeas (10%), may effectively improve spots caused by melasma [26]. However, research on this plant's ability to reduce pigmentation remains limited.

### 3.4 Sorrel

A study comparing the effects of sorrel and hydroquinone revealed that using a 3% sorrel cream for 12 weeks can significantly clarify and reduce the color of melasma spots. The study utilized Mexameter and the MASI (Melasma Area Severity Index) score to compare the sorrel and hydroquinone groups. From weeks 2 to 6, the hydroquinone group showed considerable improvement, surpassing the sorrel group. However, in the week 8 evaluation, the sorrel group exhibited a higher decrease in the MASI score compared to the hydroquinone group [27]. Nevertheless, there is a scarcity of studies on the effects of sorrel, and the exact mechanism of its impact remains unclear. It is believed that sorrel reduces pigmentation through its anti‐inflammatory activity and inhibition of tyrosinase.

### 3.5 *Aloe vera*

Numerous studies have proven the anti‐inflammatory, anti‐scar, lightening, and free radical‐reducing properties of *Aloe vera* [48]. However, when compared to phenylalanine 2%, the effect of this plant did not show significant results [28]. Additionally, evidence suggests that the using gel encapsulated with liposomes is more effective than using the gel alone [49]. The plant's impact on reducing pigmentation and its precise mechanism of action require further research.

### 3.6 Parsley

In traditional Persian medicine, parsley has long been recognized for its skin lightening and anti‐stain properties [50]. Several clinical trials have also confirmed the herb's ability to lighten the skin [51, 52]. It appears that parsley is effective in treating melasma by exerting an anti‐pigmentation effect. The study conducted by Shahla Khosravan et al. showed that this plant affects melasma‐induced spots; however, its effect is equal to hydroquinone 4% [50].

### 3.7 Licorice

Licorice is one of the most widely used plants in traditional medicine and is complementary to the treatment of skin diseases [24]. Numerous studies have confirmed its lightening and antioxidant effects. However, two specific studies found that the plant's effectiveness was higher than a placebo but lower than a hydroquinone 4% cream [24, 25, 29]. Another significant study revealed that when topical treatment of licorice is combined with 5% ascorbic acid, the recovery rate of patients significantly increases [31].

### 3.8 Tomatoes

Ancient texts have mentioned tomatoes as a natural remedy for lightening the face and reducing dark spots. Recent clinical trials have shown that patients who took tomato supplements alongside standard melasma treatment (hydroquinone 4%) experienced a greater improvement in their condition [30].

Another study found that using this plant in conjunction with rice bran also improved melasma‐induced blur. The reduction of the MASI score in the group that used the topical combination of tomato lycopene and rice bran was significantly higher than in the placebo group. Moreover, the reduction in size of the melasma lesions was significantly greater in the patients in the intervention group in comparison with the patients in the other group of the study. However, 1 month after discontinuation of this topical compound, the severity of the disease did not show any difference from the control group [36]. The level of superoxide dismutase (SOD) went up in a more recent study by Avianggi et al. when tomato lycopene extract supplements were added to sunscreen and hydroquinone 4% cream. This addition further improved the MASI scores of patients in the treatment group, showing a significant difference from the control group that only received sunscreen and hydroquinone 4% cream [38].

### 3.9 Tropical Fern

While there have been few studies on the effect of tropical fern on melasma lesions, one clinically conducted study examined the quality of life and severity of melasma in patients who received oral supplementation of tropical fern alongside standard treatment. The results of this study also indicated the effectiveness of tropical ferns in improving melasma symptoms. A comparison of the results of treatment in the follow‐up of patients showed that the group that used tropical fern supplements responded more quickly to treatment. However, in this study, the mechanism of action and active substance of this plant were not determined [32].

Moreover, 33 patients participated in another trial study, where 17 patients received a placebo three times a day and 17 patients received an equatorial fern supplement (240 mg) three times a day. The authors evaluated melanin, MASI score, and the quality of life index after 12 weeks. At the end of the 12th week, both groups showed significant improvements in the melanin standard index (placebo group 13.8% and intervention group 28.8%); however, there was no statistically significant difference between the two groups. Similarly, the MASI score in both groups decreased during the 12 weeks, but there was no difference between the two groups. Therefore, according to the results of this study, the effect of tropical fern on melasma is similar to that of a placebo [34].

### 3.10 Olive

A study has investigated the effects of both topical and edible olive oil on melasma. The study involved 42 women with melasma, divided into three groups. The first group received oral and topical placebos; the second group received an oral placebo and topical olive oil; and the third group received oral olive oil and topical placebos. We measured the melanin index and mMASI (modified Melasma Area Severity Index) score in all groups at the start of the study, and again after 60 days. The results showed that although the group treated with edible olive oil showed greater improvement, there was no significant difference between the oral and topical application of olive oil and the placebos. However, using edible olive oil for 60 days did significantly reduce the melanin index and mMASI score [33].

### 3.11 French Sea Pine Bark

In a study, pycnogenol extracted from the pine bark was orally evaluated in patients with melasma. The study included the standard three‐drug topical treatment for melasma patients and sunscreen, with the addition of pycnogenol in the intervention group. The results showed that this supplement effectively improved the quality of life for melasma patients and had a positive impact on the severity of melasma. It seems that the antioxidant properties in the extract of this plant have caused this efficacy [35].

### 3.12 Indian Gooseberry

A trial study also studied the effect of this plant in combination with licorice on blurs caused by melasma disease. 50 patients participated in this study, with 23 in the hydroquinone 2% group and 27 in the 7% Indian gooseberry and licorice intervention group. The overall results of this study showed that the combination of this plant with licorice compared to 2% hydroquinone was not significantly different in the treatment of melasma patients [37].

### 3.13 Milk Thistle

The silymarin extract from milk thistle was studied in a split‐side trial. 25 patients who had epidermal or mixed‐type melasma were selected. In this regard, 1.4% silymarin cream was applied on one side of the patient's face and 2% hydroquinone on the contralateral side for 3 months. mMASI scores decreased on both sides but were only statistically significant on the hydroquinone side, which caused a 17.97% reduction vs. only 7.11% on the silymarin side. No statistical difference in mMASI was found between the two groups [39].

### 3.14 Lentil, Bitter Almond, and Fig (Tila‐e‐Kalf)

Tila‐e‐Kalf is an Unani pharmacopoeial formulation used in a clinical trial for treating melasma. This remedy is made from a mixture of lentil and bitter almond powder, which is then added to the decoction of fig to form a homogenous paste. The control group received 4% hydroquinone. Both treatment and control groups were advised to apply sunscreen twice a day. This study revealed that Tila‐e‐Kalf has the same effect as hydroquinone. Only one patient treated with Tila‐e‐Kalf experienced mild itching, whereas the patients treated with hydroquinone cream reported more side effects [40].

### 3.15 *Dorema ammoniacum*

*Dorema ammoniacum* is an herb native to Iran with anti‐inflammatory effects. In the study conducted by Javedan et al., 49 patients were separated into control and drug groups; they received placebo and the drug, respectively, twice a day for 30 days. Javedan et al. administered the remedy in the form of a 6% topical gum. The results of this study demonstrated that *Dorema ammoniacum* was significantly more effective in treating melasma in comparison with placebo [41].

### 3.16 Multi‐Herbal Syrup Containing Lemon Balm, Damask Rose, and Fenne

Lemon balm is a natural remedy with antibacterial and anti‐inflammatory effects that is effective in skin rejuvenation [53, 54]. Damask rose is also effective in skin rejuvenation, with effects on hydration, improvement of stretch marks and wrinkles, acne control, and pigmentation reduction [55, 56]. Fennel possesses anti‐inflammatory and anti‐melanogenesis properties, making it an effective treatment for both digestive issues and skin diseases [57, 58, 59]. A randomized, triple‐blind, placebo‐controlled clinical trial was done with 110 patients (12 weeks) to see how well this multi‐herbal syrup treated melisma. The intervention group had 55 and the placebo group 55. Parameters including melanin, lightness, pigmentation, and the Melasma Quality of Life (MELASQOL) were significantly different in comparison to placebo. Therefore, this multi‐herbal syrup is an effective treatment for melisma [42].

### 3.17 *Terminalia chebula* Retz

*Terminalia chebula* Retz. ( *T. chebula* ), native to India and Southeast Asia, is most important and is used orally and topically in traditional medicine. *T. chebula* is a beneficial choice for treating melisma due to its antioxidant, anti‐inflammatory, and tyrosinase enzyme inhibitory activities. In a randomized, controlled, triple‐blind clinical trial, one group received *T. chebula* 5% cream and the other hydroquinone 2% cream at bedtime for 12 weeks. *T. chebula* groups significantly reduced their mMASI score 4, 8, and 12 weeks after initiating the study. The *T. chebula* group significantly reduced skin irritation compared to the hydroquinone group [43].

## 4 Discussion

Even though there are various therapies available for melasma, none of them guarantee a satisfactory result, and the management of this condition remains a challenge. Humans have used herbal medicine for centuries, and it may hold the key to many of modern medicine's mysteries. We aimed to review the articles that examined the efficacy and safety of various herbal drugs in the treatment of melasma.

With this goal in mind, a total of 21 articles were reviewed and evaluated by type of plant. No patient had any severe reactions to the herbs, and only seven patients had mild reactions to some of the herbal medications used (parsley, licorice, rhubarb, and Tila‐e‐Kalf), which could argue for the safety of these herbs [24, 37]. As observed, it seems that parsley, licorice, sorrel, chickpeas, tropical fern, tomato, pine bark (pycnogenol), *Dorema ammoniacum* , melon seed, Tila‐e‐Kalf (lentil, bitter almond, and fig), *T. chebula* , and a polyherbal syrup containing Lemon Balm, Damask Rose, and Fennel had effects to some extent on melasma‐induced stains [24, 27, 33, 41, 50].

Licorice caused significantly better results than placebo but was less effective than hydroquinone cream [24, 25]. Researchers also discovered that combining 4% licorice with 5% ascorbic acid led to a more significant improvement compared to using 4% licorice alone [31]. Another study compared a mixture of licorice and gooseberry with 2% hydroquinone cream and found no significant difference between them in treatment of melasma [37]. Studies have shown that licorice with a melanin dispersibility mechanism can lead to depigmentation. In the study of Yokota and his colleagues and Matoorianpour et al., it has also been shown that licorice reduces melanocyte activity and melanin production due to glabridin (a flavonoid compound), which reduces pigmentation in patients. It seems that this plant can be effective in improving this disease by reducing melanocyte production [60]. Researchers have reported using licorice to treat skin pigmentation because of its tyrosinase inhibitory activity. Therefore, compounds that function as tyrosinase inhibitors have a significant impact on cosmetic items as agents that lighten the skin and in the management of certain dermatological conditions. Furthermore, flavonoids constitute the predominant category of natural phenolic compounds that function as inhibitors of tyrosinase. Licorice extracts and three main flavonoids—licochalcone A, glabridin, and dehydroglyasperin C—are the compounds that have been studied the most in dermatology for their skin‐whitening and anti‐aging effects [61, 62, 63, 64].

Evidence showed that a mixture of chickpeas and melon seeds could be an acceptable herbal regime for the treatment of patients with melasma. In this context, the Iranian population commonly uses chickpea as a drug and food, prompting several studies to investigate its therapeutic effects [26, 65, 66]. Evidence demonstrated that chickpeas have anti‐tyrosinase and antioxidant activities, which could potentially have anti‐hyperpigmentation and skin‐whitening effects [67].

Sorrel was compared to hydroquinone cream; the standard treatment showed better improvements from the 2nd week until the 6th, but after 8 weeks of treatment, the sorrel group showed significantly better results. The sorrel plant reduces melanin production with a tyrosinase inhibition mechanism and thus effectively improves hyperpigmentation disorders such as melasma [27, 60]. In addition, parsley had similar results to hydroquinone cream [50]. Tomato supplements (lycopene extract) also caused a significant improvement in patients' disease compared to placebo. It was also efficient at increasing the benefits of routine treatment with sunscreen and 4% hydroquinone by increasing the levels of SOD [30, 38].

Evidence revealed that some plants, such as tomatoes, sorrels, and parsley, have antioxidant properties and could improve the severity of melasma disease due to the reduction of free radicals [36, 68]. Especially in patients with melasma, sunlight exposure caused more severe symptoms. These plants could also have preventive properties for these susceptible individuals [30, 36]. Despite the significant effects of some of these plants on melasma, even compared to the current standard of treatment drugs, there are few studies in this field, and only a few clinical trials have been conducted on these plants and their effects on skin darkening caused by melasma.

A combination of 0.05% lycopene extracted from tomato and 3.45% wheat bran topical use showed improvements in pigmentation while using the treatment, but 1 month after discontinuation of the drug, no significant difference was seen between treated patients and those receiving placebo [36]. Tropical fern supplements showed mixed results compared to placebo; one study found significance compared to placebo, and the other found no difference [32, 34]. The pine bark pycnogenol supplement helped improve the outcome in patients receiving the standard three‐drug treatment, probably due to its antioxidant properties [35].

Lentil, bitter almond, and fig mixture (Tila‐e‐Kalf) had the same effect as 4% hydroquinone cream with fewer side effects [29]. *Dorema ammoniacum* was also shown to be significantly better than placebo, but the conclusion cannot be relied upon due to the randomization issue in the study [41]. Rhubarb, *aloe vera* , silymarin extract from milk thistle, and olive showed no significant improvement in patient outcomes [28, 33, 39]. Although edible olive oil use for 60 days did reduce the melanin index and mMASI score, it had no significant difference from the placebo and topical olive oil groups during 90 days of administration [33]. However, the authors recommend designing similar studies on these herbal remedies for the treatment of melasma and other skin hyperpigmentation conditions, using different doses, shapes, formulations, and concentrations of the cosmetic products or medicine.

Traditional Persian medicine texts like Bu‐Ali Sina Law, Al‐Hawi Razi, Reservoir al‐Adouyeh, and the Book of Akbari Medicine list several plants with anti‐pigmentation properties. This text suggests saffron as a plant that reduces facial skin stains and mentions it as a treatment for melasma [69]. The active ingredients of the saffron plant are crocetin and carotenoids, which prevent the production of free radicals in the skin [70]. Researchers have also mentioned *fumaria parviflora* and leek plants as skin protectors against ultraviolet radiation and sunlight, thanks to their phenolic and ascorbic acid compounds. Researchers have also reported their effectiveness in treating melasma by reducing stain and lightening the skin in these patients [9]. Other plants, such as fenugreek, grapes, figs, and celery, were mentioned in Iranian medicine texts. They have the ability to lessen the darkening resulting from melasma and shield the skin from intensifying these stains [69]. It is also stated that the compounds affecting melasma in these plants include phenolic, carotenoid, crastin, ascorbic acid, tannins, tyrosinase inhibitors, and anti‐inflammatory compounds [37].

Previous research has not clearly stated the mechanisms of action of some herbal remedies presented in this scoping review study. However, it looks like the tyrosinase inhibitors, retinoid, and anti‐inflammatory compounds in these plants are crucial in reducing pigmentation in these patients [48, 71, 72, 73, 74]. In addition, phytochemicals may mitigate the detrimental effects of UV radiation on human skin. Phenolic compounds constitute the most significant category of phytochemicals that can function as sunscreen agents. In this regard, evidence suggests that apigenin and chlorogenic acid, two phenolic compounds, are the most effective UVB and UVA filters for treating skin hyperpigmentation and preventing its onset or exacerbation [75, 76]. Several studies have also shown that secondary metabolites found in plants can change the way melanocyte cells communicate during the melanogenesis process. Regarding this, numerous studies have focused on the regulation of the MC1R, c‐KIT, WNT, and ETBR pathways, which are essential in modulating skin pigmentation and melanocyte production [75, 77, 78].

This study was subject to several limitations. First, the authors focused on the published articles for inclusion in the study, so the gray literature was not investigated in this study. Second, there were few studies on the treatment of patients with melasma, so because of the methodological heterogeneity of the studies and the low number of studies, conducting a meta‐analysis was not applicable. Then, the risk of bias assessment of the included articles was not considered in this study, so the authors recommend considering this issue in future studies. Next, a network meta‐analysis to compare the efficacy of different herbal medications in treating patients with melasma was not the goal of this study; the authors suggest this issue in further studies. Finally, the phytochemical components and mechanism of action of these herbal remedies in threating melasma and skin hyperpigmentation were not definitely discovered. Therefore, we recommend further studies to unravel these properties.

## 5 Conclusion

Despite the antioxidant, anti‐tyrosinase, and illuminating properties of many medicinal plants and the several recommendations of traditional Persian medicine sources on the effect of these plants, few studies have investigated the effect of these plants on melasma‐induced skin spots. In addition, the existing evidence about the effects of these plants is still inadequate, and further studies with larger sample sizes are needed to confirm the effects of these plants.

## Author Contributions

M.M.P. and N.S. designed the study. M.M.P., N.Y., A.M., M.H., and R.J. collected the data. M.M.P., R.J., M.H., N.Y., and A.M. drafted the manuscript. M.M.P., N.S., and M.H. finalized the manuscript. All authors reviewed the manuscript and approved the final version. They take full responsibility for the content and writing of this article.

## Ethics Statement

The protocol of this study was approved by the Ethics Committee of Shiraz University of Medical Sciences (Ethics code: IR.SUMS.MED.REC.1399.597).

## Conflicts of Interest

The authors declare no conflicts of interest.

## Data Availability

The data that support the findings of this study are available on request from the corresponding author. The data are not publicly available due to privacy or ethical restriction.
